# Diphtheria and Its Treatment

**Published:** 1878-11-20

**Authors:** H. A. Eberle


### DIPHTHERIA AND ITS TREATMENT.

BY H. A. EBERLE, M. D., C. M.

In this region of Iowa we have had to battle with the above dreaded disease for two years, and ever since its invasion there have been many large families of children entirely swept away with the disease. The profession have put forth more than ordinary exertions in trying to discover a mode of treatment that would match its deadly ravages, and, I believe, with partial success. In this communication, we do not intend to discuss the pathology of a disease on which so many scientific authors differ, and to which Oertel has done such masterly justice in giving so clear a description of its morbid condition; but if we could aid the profession in any manner in giving our experience and sucess with the plan of treatment adopted, we would be doing nothing but our duty.

On August 30th, 1878, we had the privi ege of seeing a girl, thirteen years old, suffering severe symptoms of diphtheria. She was the sixth child in a family of eight that was smitten with the malady, five having previously taken the disease, and all died, under the care of Dr. Medbery, of this city. Dr. M., observing the case in the morning, and knowing it to be a severe one, remarked that he could "do nothing for her"; that "she would die, as the others had." The family, not feeling satisfied with this prognosis, sent in haste, and I was soon on the scene of action. Ascertaining that the bowels were locked up for several days, I ordered magnes. cit., one tablespoonful, at once. I then prescribed the following: R. Sodæ sulphocarbolatis, grs. xxx; sacch. lactis, grs, xxx; m. ft. divide into xx powders. Sig.: one powder to be taken every hour. Also, the following as a gargle: R. Pulv. potas chlor., ʒ iij; acidi hydrochloric dil., ʒij; tinc. capsici. ʒj; aqua ad., q. s. ℥viij. M. S.—Use as a gargle every half hour.

In connection with this treatment, I adopted a novel plan of anointing the patches of micrococci with a steam atomizer, throwing a solution of carbolic acid, alcohol and water, of sufficient strength, on the tonsils, throat and nasal passages, by inhaling. The plan pursued worked like a charm, for, on my next visit, I was delighted to see no increase in the disease, and the temperature had abated to 101° F., which was 103.5° the day before.

O■ September 1st, my patient was convalescing, the powders were lessened to intervals of three hours, the steaming also used every three hours.

Patient No 2, a girl, nineteen years of age, in the same family, sickened on the 5th of September. Her initial symptoms were very severe. Temp., 104° ; Pulse, 120°. The same plan of treetment was adopted. The only difference was an increase in the amount of medicine which was three grains of the sulpho-carbolate of soda every hour ; the gargle and atomizer used as in the preceeding case. In three days my patient was convalescent.

Family No. 2.—in this family the worst features of the disease was manifest. There were nine children in the family, and eight, whose ages averaged between 18 months and 16 years, took the disease one after the other. The family, being German, the house small, and the comforts few, while the sanitary condition was anything but good, I anticipated a large mortality.

Patient 6, in this family, a chubby little boy of three years, took the disease, when at the same time he had a severe coryza. I remarked to the parents that this one could not surely get well, but, after four days of careful treatment, he began to amend ; when the mother "stepped out" a moment, the little fellow bounded out on the floor, barefooted, and crept to the door. Before he could be put back to bed, he had "taken cold," and at my next visit I found fresh invasions of the disease. The nasal passages were attacked, and the pharynx completely filled with the cryptogamic products. Ammon. mur., in grain doses, was added to the carbolate, and stimulants given freely, but nothing could stay the progress of the disease, death taking place the following day by suffocation.

Since the commencement of the above plan of treatment, I have treated thirteen cases, and one of which died. I attribute my success partly to non-interferance, locally, with swabbings, or painting the pseudo-membrane, which, in the hands of the most expert physicians, especially where small children are the patients, is a dangerous procedure; and, when this part of the programme is left to nurses or parents, the tender and gorged mucous membrane is sure to be wounded, which causes at once a fresh surface for the bacteria to infest, and. moreover, small abscesses from beneath the mucosa and submucosa, the products of which are readily absorbed into the circulation, causing general blood poisoning.—*Michigan Medical News.*

